# Medium Spiny Neurons Mediate Timing Perception in Coordination with Prefrontal Neurons in Primates

**DOI:** 10.1002/advs.202412963

**Published:** 2025-02-11

**Authors:** Xinhe Liu, Zhiting Zhang, Lu Gan, Panke Yu, Ji Dai

**Affiliations:** ^1^ Shenzhen Technological Research Center for Primate Translational Medicine Shenzhen‐Hong Kong Institutes of Brain Science Shenzhen Institutes of Advanced Technology Chinese Academy of Sciences Shenzhen 518055 China; ^2^ CAS Key Laboratory of Brain Connectome and Manipulation the Brain Cognition and Brain Disease Institutes Shenzhen Institutes of Advanced Technology Chinese Academy of Sciences Shenzhen 518055 China; ^3^ Guangdong Provincial Key Laboratory of Brain Connectome and Behavior Shenzhen Institutes of Advanced Technology Chinese Academy of Sciences Shenzhen 518055 China; ^4^ Research Center for Medical Artificial Intelligence Shenzhen Institutes of Advanced Technology Chinese Academy of Sciences Shenzhen 518055 China; ^5^ University of Chinese Academy of Sciences Beijing 100049 China

**Keywords:** D2‐MSNs, medium spiny neurons, neural trajectory, perception, prefrontal cortex, striatum, timing

## Abstract

Timing perception is a fundamental cognitive function that allows organisms to navigate their environment effectively, encompassing both prospective and retrospective timing. Despite significant advancements in understanding how the brain processes temporal information, the neural mechanisms underlying these two forms of timing remain largely unexplored. In this study, it aims to bridge this knowledge gap by elucidating the functional roles of various neuronal populations in the striatum and prefrontal cortex (PFC) in shaping subjective experiences of time. Utilizing a large‐scale electrode array, it recorded responses from over 3000 neurons in the striatum and PFC of macaque monkeys during timing tasks. The analysis classified neurons into distinct groups and revealed that retrospective and prospective timings are governed by separate neural processes. Specifically, this study demonstrates that medium spiny neurons (MSNs) in the striatum play a crucial role in facilitating these timing processes. Through cell‐type‐specific manipulation, it identified D2‐MSNs as the primary contributors to both forms of timing. Additionally, the findings indicate that effective processing of timing requires coordination between the PFC and the striatum. In summary, this study advances the understanding of the neural foundations of timing perception and highlights its behavioral implications.

## Graphical abstract

Timing perception is a crucial cognitive function enabling effective navigation in the environment, involving both prospective and retrospective timing. This study investigates the neural mechanisms in monkeys, uncovering distinct processes that shape temporal perception and highlighting the essential role of D2 medium spiny neurons in the striatum as key facilitators of timing processes.

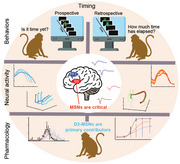

## 1 Introduction

Time perception is a fundamental cognitive ability that allows individuals to navigate and interact with their environment effectively. In particular, two major forms of timing have been identified: prospective timing, which involves anticipating the duration of upcoming events, and retrospective timing, which pertains to the evaluation of the duration of completed events.^[^ ^1^ ^]^ Understanding the neural mechanisms underlying these two forms of timing is crucial for a deeper appreciation of how the brain processes temporal information.

The prefrontal cortex (PFC) is widely recognized for its critical role in managing attention and cognitive control, while the basal ganglia plays a key role in integrating timing with actions and reward mechanisms. Numerous studies have been conducted to investigate the functional relationships between these regions and their involvement in timing. Evidence from previous research indicates that both the PFC and the striatum are engaged in prospective timing as well as retrospective timing.^[^ ^2^ ^]^ Within the cortical‐striatal circuitry, neurons display a range of encoding patterns during timing processing, which include: 1) “ramping coding,” where neurons exhibit a linear increase in firing rates throughout the timing task;^[^ ^2^, ^3^ ^]^ 2) “peak coding,” characterized by groups of neurons that reach their firing peaks at sequential moments during the timing process;^[^ ^2^, ^3^, ^4^ ^]^ 3) “absolute coding,” in which neurons respond similarly regardless of the duration being timed;^[^ ^3^, ^4^, ^5^ ^]^ and 4) “relative coding” or “scalar coding,” where neuron firing patterns adapt—either compressing or extending—according to the lengths of different durations.^[^ ^2^, ^5^, ^6^ ^]^ Collectively, these insights enhance our comprehension of how temporal information is processed within the brain.

Despite an increasing understanding of the roles played by the striatum and prefrontal cortex in timing processes, substantial knowledge gaps persist. Notably, the dynamics of how these neural mechanisms collaborate across different timing tasks remain elusive, as examines the interplay between the striatum and prefrontal cortex in both prospective and retrospective timing scenarios. Furthermore, the distinct functions of different neuronal populations within these areas, particularly the medium spiny neurons (MSNs) found in the striatum, have yet to be thoroughly investigated. As a crucial component of the basal ganglia, the striatum contains a wide range of neuronal populations that may uniquely influence the perception of time.

In the present study, we aimed to address these gaps by clarifying the functional roles of various neuronal populations in shaping subjective experiences of time. To achieve this, we utilized single‐unit recordings to investigate the neuronal activity of thousands of neurons in the striatum and prefrontal cortex of macaque monkeys during timing tasks. This research seeks to deepen our understanding of how these interconnected brain regions contribute to temporal processing and to tackle key questions about the neural basis of time perception. Ultimately, our findings have the potential to offer valuable insights into the broader cognitive mechanisms involved in time perception, which could inform future research on decision‐making, learning, and memory.

## 2 Results

The results are structured as follows: First, we confirm that monkeys exhibit accurate abilities in both retrospective and prospective timing, as anticipated. Next, we detail the classification of neurons and their collective responses to timing, emphasizing their unique characteristics in relation to retrospective and prospective timing. Following this, we explore the roles of “absolute coding” and “scalar coding” neurons, particularly highlighting the significant role of MSNs in timing mediation through an analysis of neural trajectories. Additionally, we establish that D2‐MSNs are the key players in both timing tasks by selectively manipulating specific cell types. Finally, we emphasize the critical role of neural coordination between the PFC and the striatum in enhancing temporal processing.

### 2.1 Monkeys Possess Precise Retrospective and Prospective Timing Ability

**Figure 1:**
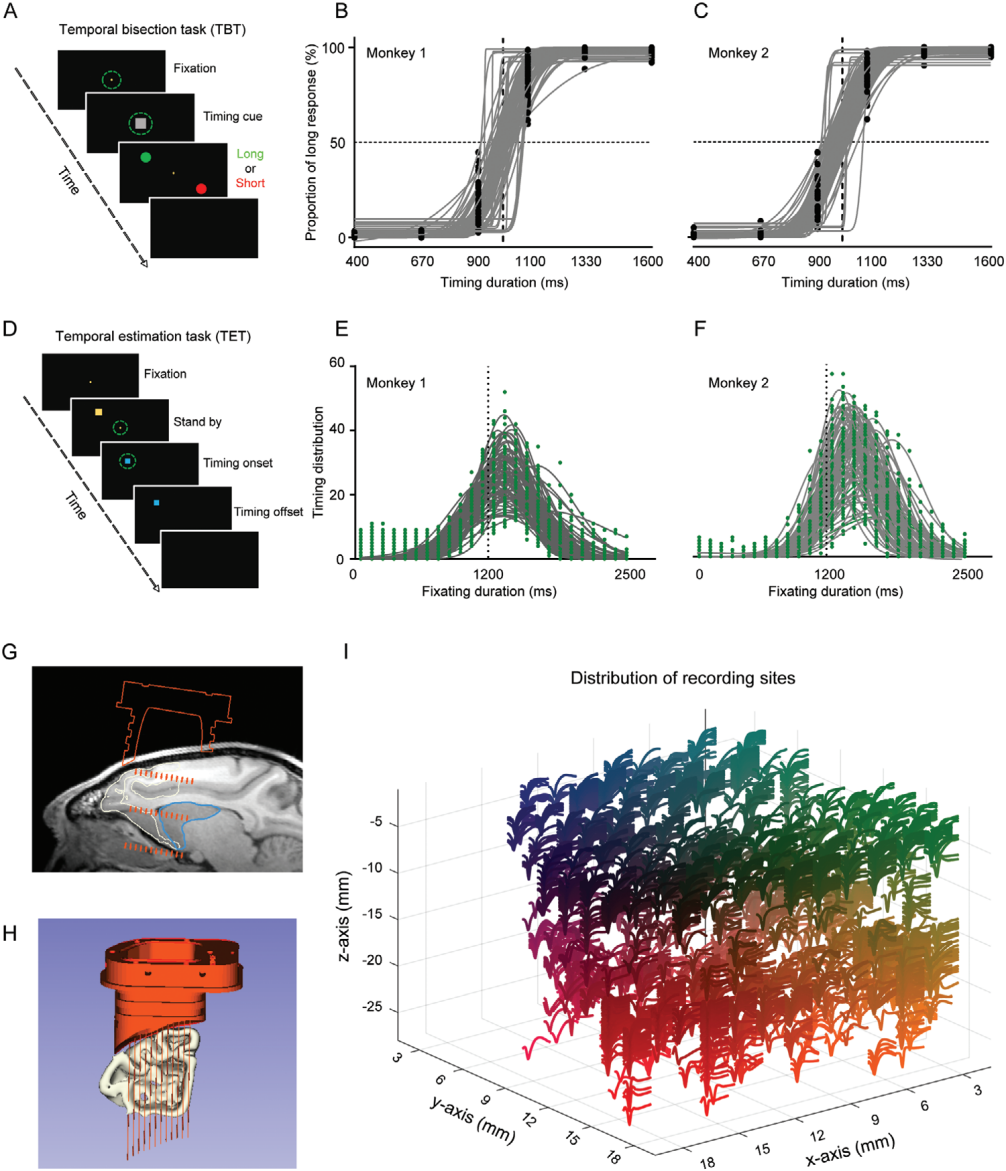
Timing Behavioral Paradigm and Single‐Unit Recording Methods. A) Schematic representation of the retrospective timing task, referred to as the Temporal Bisection Task (TBT). B,C) Behavioral performance of two monkeys during the TBT, measured as the proportion of trials in which the monkeys chose “long” for each specific timing duration (y‐axis). D) Schematic overview of the prospective timing task, known as the Temporal Estimation Task (TET). E,F) Behavioral performance of the same two monkeys in the TET; the distributions of estimated timing are fitted with a Gaussian function. G,H) Illustration of the recording sites shown in MRI scans, with the reconstructed electrode array overlaid on the brain's anatomy. I) Distribution of all recording sites alongside their respective recorded spiking waveforms. The use of gradient colors serves to improve the readability of the different waveforms.

In the current study, two rhesus monkeys were trained to engage in tasks assessing both retrospective and prospective timing: the temporal bisection task (TBT, **Figure** **1**A; Video S1, Supporting Information) and the temporal estimation task (TET, Figure 1D; Video S2, Supporting Information).^[^ ^7^ ^]^ During the TBT, the subjects needed to judge whether the duration of a visual stimulus was longer or shorter than 1 s. Analysis of their responses indicated that performance on the TBT closely matched a cumulative Gaussian function (Figure 1B,C). In the TET, participants were asked to estimate a duration of 1.2 s, with their results also fitting a Gaussian distribution (Figure 1E,F). Notably, both tasks exhibited a rightward shift in the average responses, measuring at 1471 and 1350 ms for monkeys 1 and 2, respectively, indicating a discrepancy from the intended duration. These findings highlight the monkeys' precise time perception within the millisecond to second range across both types of timing tasks.

During the execution of these behavioral tasks, we recorded extracellular activity from the striatum and PFC using a 128‐channel semi‐chronic electrode array (Figure 1G–I, see Experimental Section for more details). To ensure accuracy in our recordings, we utilized magnetic resonance imaging (MRI) and post‐surgical computed tomography (CT) scans for site validation. Ultimately, 3511 well‐isolated neurons were identified. Of them, 1333 in the striatum and 1944 in the PFC were included in the following analysis.

### 2.2 Populational Responses to Retrospective and Prospective Timings Display Distinct Features

**Figure 2:**
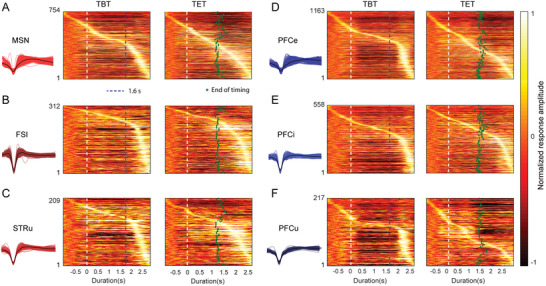
Classification of Neuron Groups and Their Responses to the Two Timing Tasks. Heatmaps illustrate the responses of various neuronal groups to the TBT and TET. Each heatmap displays the activity of all neurons within a specific group during the respective task. The number of neurons in each group is indicated on the left side of the heatmap, while the corresponding spiking waveforms are shown beneath each group label. A–C) Neuronal responses from medium spiny neurons (MSN), fast‐spiking interneurons (FSI), and unidentified units (STRu) in the striatum. D–F) Neuronal responses from prefrontal cortex ensembles, including excitatory neurons (PFCe), inhibitory neurons (PFCi), and unidentified neurons (PFCu) in the prefrontal cortex.

We began by categorizing all neurons according to their origin, distinguishing between those from the striatum and the PFC. Subsequently, we employed a dimensionality reduction technique to classify the neurons based on their waveforms,^[^ ^8^ ^]^ resulting in three clusters for both the striatum and PFC. Each cluster was then associated with a putative neuron type, informed by their waveform characteristics and response properties. For striatal neurons, MSNs emerged as the predominant cell type, leading us to categorize regular spiking neurons as MSNs (**Figure** **2**A; Figure S1, Supporting Information). Neurons exhibiting narrow peaks and rapid spiking rates were classified as fast‐spiking interneurons (FSIs, Figure 2B), while the remaining undefined striatal neurons were grouped as STRu (Figure 2C). In the case of PFC neurons, those with regular peaks were identified as excitatory neurons (PFCe, Figure 2D), narrow peaks were designated as interneurons (PFCi, Figure 2E), and the remainder fell into the undefined category (PFCu, Figure 2F).

Following this classification, we calculated the firing rates of each neuron in response to the TBT, covering 1600 ms trials, as well as the TET. We illustrated these normalized firing rates in a heat map format, with each row representing a neuron subgroup aligned to the stimulus onset and sorted by the peak response time, resulting in a continuous bright band within each heat map. These visualizations provided insight into the distinct characteristics exhibited by various cell types in relation to retrospective and prospective timing.

Analysis of the peak responses within these bands revealed that the neuronal processing for retrospective and prospective timing represents two separate mechanisms. For MSNs, the percentage of neurons that exhibited peak activity during timing was similar across both tasks, at 19.76% and 22.41% (**Table** **1** and Figure 2A). In contrast, the proportions for PFCe neurons varied significantly; 11.35% peaked during TBT, while 21.93% did so during TET (Table 1). Among inhibitory neurons, the proportions for FSI were 12.50% and 7.05% respectively, while PFCi neurons displayed comparable percentages of 12.54% in TBT and 13.98% in TET, suggesting that interneurons in the striatum and PFC may assume differing roles in the timing processes. Meanwhile, the undefined category PFCu demonstrated a response pattern akin to PFCe, whereas STRu neurons exhibited a response pattern more in line with FSI. Collectively, these findings highlight the variability among neuron types and regions in their roles in the processing of retrospective and prospective timing.

**Table 1:** Percentage of neurons showing different features in each group.

Type	Number	Peaked during timing		Absolute coding in TBT	Scalar coding in TET	Absolute in TBT & Scalar in TET	Responded similarly to TBT & TET
		TBT	TET				
MSN	754	19.76%	22.41%	33.42%	16.84%	11.67%	20.42%
FSI	312	12.50%	7.05%	8.97%	4.81%	2.24%	8.01%
STRu	209	16.27%	9.09%	18.66%	7.18%	3.83%	9.57%
PFCe	1163	11.35%	21.93%	20.12%	6.28%	2.92%	7.65%
PFCi	558	12.54%	13.98%	7.71%	3.94%	1.25%	8.78%
PFCu	217	9.22%	25.35%	27.65%	10.60%	5.53%	5.99%
Striatum	1333	17.25%	16.20%	24.91%	12.23%	7.80%	16.13%
PFC	1944	11.47%	19.96%	17.39%	6.07%	2.73%	7.87%
Total	3277	13.82%	18.43%	20.45%	8.57%	4.79%	11.23%

### 2.3 Retrospective and Prospective Timings Employ Distinct Coding Strategies

**Figure 3:**
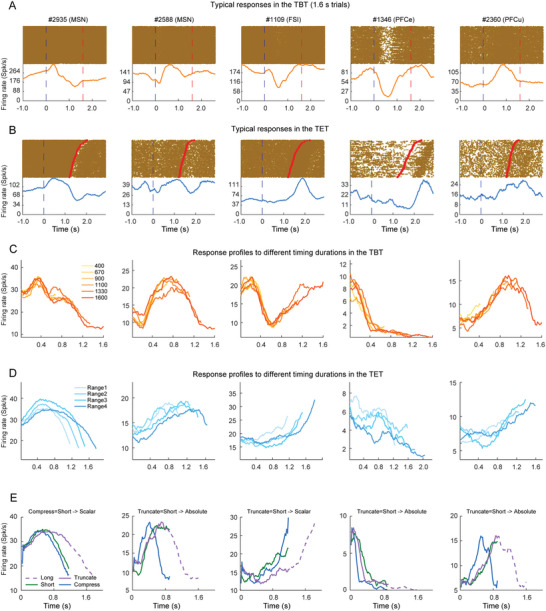
Typical Responses of Five Example Neurons during the TBT and TET. A,B) Raster plots and accumulated response profiles are shown for both the TBT and TET. The blue and red dashed lines mark the onset and offset of the timing intervals, respectively. C) Response profiles for six distinct timing durations in the TBT. D) Response profiles for four specific timing ranges in the TET. E) The responses of these neurons were classified into “scalar coding” or “absolute coding” categories based on their activity patterns in response to short versus long durations.

The considerable variation observed among different cell types in processing retrospective and prospective timing led us to investigate the presence of a common mechanism underlying these two timing processes. We examined the response characteristics of specific neurons engaged in distinct timing tasks. Initially, we presented typical response profiles from three striate and two PFC neurons in responding to the TBT (**Figure** **3**A, including only 1600 ms trials) and TET (Figure 3B). To assess the degree of similarity between these two timing conditions, we calculated the correlation coefficient (r) between neuronal responses to TBT and TET, yielding values of r = 0.33, 0.53, 0.33, 0.34, and 0.70 for each pair. By applying a threshold of r > 0.5 (*p* < 0.001), we identified that only 11.23% of neurons employed comparable processing strategies for TBT and TET (Table 1). The low percentage of neurons responding similarly to the two forms of timing imply that the brain might take different coding mechanisms when processing retrospective and prospective timing. Interestingly, 20.42% of MSNs fell into this group, contrasted with 8.01% of FSI and 7.87% of PFC. This elevated proportion of MSN exhibiting similar responses to both timing forms underscores their significance in temporal processing.

Subsequently, we aimed to understand how varying time lengths are represented at the neuronal level, prompting us to compare response profiles across six timing conditions in the TBT (Figure 3C). For the TET, because timing durations were not predetermined and instead were reliant on the subjects (Figure 1E,F), a broad spectrum of durations emerged. Consequently, we categorized the trials into four ranges, from the shortest to the longest durations. The response profiles of these neurons to the timing lengths in the TET are depicted in Figure 3D. The neuronal responses revealed nearly identical patterns across the different time lengths in the TBT, indicating an “absolute coding” modality for retrospective timing.^[^ ^4a^ ^]^ Conversely, in the TET, certain neurons demonstrated scalable response profiles where the reaction to shorter durations appeared to be a scaled version of responses to longer durations (indicative of scalar coding), exemplified by neuron #2935 in Figure 3D. To classify neurons, we either downscaled or truncated the response profile of the longest duration and then compared it with that of the shortest duration. A neuron was designated as employing absolute coding if its response to the shorter duration closely matched the truncated profile; those matching the scaled‐down response were classified as scalar coding (Figure 3E, with further details available in the Experimental Section). Based on these criteria, neurons #2935 and #1109 were identified as scalar coding neurons within the TET, whereas neurons #2588, #1346, and #2360 were categorized as absolute coding neurons in the TBT. Our findings revealed that in the TBT, 20.45% of neurons took the absolute coding strategy, with only 0.18% took scalar coding. In contrast, in the TET, 8.57% of neurons took scalar coding and only 0.52% took absolute coding. This divergence highlighted the contrasting modes of encoding for retrospective and prospective timing: absolute coding primarily for TBT and scalar coding for TET. Notably, 33.42% of MSN were designated as absolute coding in the TBT, while 16.84% functioned as scalar coding in the TET, with 11.67% of these neurons displaying both characteristics simultaneously (Table 1). These absolute or scalar coding MSNs were primary distributed in the medial site of the striatum, suggesting the functional relevance of the caudate in timing (Figure S2, Supporting Information).

### 2.4 MSNs are Critical for Both Retrospective and Prospective Timings

**Figure 4:**
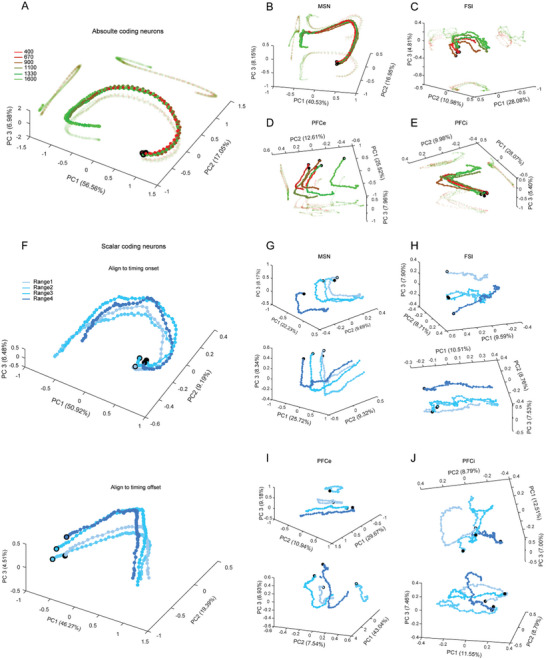
Neural Trajectories of Different Neuron Groups During Timing Tasks in the TBT and TET. A) Neural trajectories of absolute coding neurons for six timing durations in the TBT. The percentage in each bracket indicate the contributing rate of each principal component (PC). B‐E) Neural trajectories of the MSN, FSI, PFCe, and PFCi groups during the TBT. F) Neural trajectories of scalar coding neurons across four timing ranges in the TET. The top and bottom plots are aligned to the onset and offset of the timing intervals, respectively. G‐J) Neural trajectories of MSN, FSI, PFCe, and PFCi groups during the TET.

In addition to analyzing the response patterns at the individual neuron level, we further elucidate the dynamics of neural responses at the population level by calculating the neural trajectories for each group of neurons. These trajectories reflect their overall dynamic changes throughout the timing process.^[^ ^2^, ^3^, ^6^ ^]^ To effectively visualize these trajectories, we projected the first three principal components (PC) into a three‐dimensional PC space. For the TBT, we initially plotted the neural trajectories of absolute coding neurons at a 20 ms resolution, which captured the primary coding features during prospective timing (**Figure** **4**A). Our analysis revealed that the trajectories for varying timing lengths demonstrated a high degree of replication once timing commenced (indicated by the black dots), with longer durations correlating with extended trajectories. This significant repeatability underscores a common coding strategy for varying durations among these absolute neurons. Nonetheless, when we examined the neural trajectories for specific cell types, we observed that such repeatability was predominantly evident in MSN (Figure 4B–E; refer to Figure S3, Supporting Information for additional undefined cell types). This finding indicates that MSN generally employs an absolute coding strategy for encoding retrospective timing, while other cell types may not utilize this approach.

For the TET, monkeys exhibited subjective timing with variability across trials, complicating the creation of a uniform data matrix as required for trajectory plotting in the TBT, since equal data lengths were necessary. To address this, we included trials that lasted between 1000 and 2400 ms and categorized them into four ranges for each recording session, with ranges 1–4 representing durations from short to long. Subsequently, we formulated neural trajectories for these ranges aligning to the timing onset or offset. Notably, we discovered that the trajectories in the TET diverged significantly from those observed in the TBT. Specifically, for the prominent neuron type—scalar neurons—in the TET, their trajectories across different subjective timing ranges demonstrated correlations, whether assessed by aligning to timing onset (top of Figure 4F) or offset (bottom). While a similar correlation pattern was identified for MSN (Figure 4G), no discernible patterns were noted for PFCe, PFCi, or FSI (Figure 4H–J; see Figure S3, Supporting Information for undefined cell types), suggesting that MSN plays a vital role in prospective timing as well.

**Figure 5:**
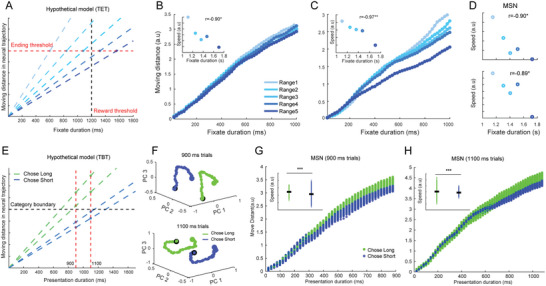
Moving Distance and Speed of Neural Trajectories for MSNs Reflect Subjective Timing. A) A hypothetical model showing the relationship between moving distance and timing duration during the TET. B,C) Cumulative moving distance for five subjective timing ranges in the TET for scalar coding neurons. Panels (B) and (C) are aligned to the onset and offset of the timing intervals, respectively. The inset displays the correlation between subjective timings and the moving speed of the neural trajectories. Asterisks indicate the significance of the Pearson product‐moment correlation. *p < 0.05, **p < 0.01. D) Correlation between subjective timings and moving speed of neural trajectories specifically for MSNs. E) A hypothetical model demonstrating the relationship between moving distance and timing duration in the TBT. F) Neural trajectories for Long and Short choice trials in the 900 and 1100 ms conditions, with green representing “Chose Long” and blue representing “Chose Short.” G,H) Cumulative moving distance for Long and Short choice trials in the 900 and 1100 ms conditions for MSNs. Insets show the moving speeds for the Long and Short choices. Statistical significance is indicated by *p < 0.05, **p < 0.01, ***p < 0.001, paired *t*‐test.

Moreover, the divergence of these trajectories prompted us to suspect whether the cumulative moving distance or overall speed of these trajectories could serve as indicators of subjective timing. We posited that a shorter moving distance for a neural trajectory over a fixed duration correlates with an extended subjective timing perception (**Figure** **5**A). To test this theory, we computed the cumulative moving distance for each neural trajectory created from primary PCs accounting for over 70% contributing rate across all timing ranges. We categorized the trials into five distinct ranges to facilitate a quantifiable relationship between subjective timing and moving distance for subsequent analysis. By graphing the accumulated distance against time for each range, we discovered that for the scalar neurons, the distances conformed precisely to our theoretical model, whether observed after timing onset (Figure 5B) or before timing offset (Figure 5C). Further calculations of the average moving speed for each trajectory revealed a strong negative correlation (r = −0.90 and ‐0.97, *p* < 0.05, insets of Figure 5B,C) with the subjects’ fixation durations across each timing range. Given that fixation duration indicated subjective time within our experimental framework, we concluded that a slower speed in neural trajectories correlates with a lengthened subjective timing for scalar neurons. Additionally, we replicated this analysis across all cell groups and discovered that only the MSN group exhibited a significant negative correlation (r = ‐0.90 and ‐0.89, *p* < 0.05, Figure 5D; see Figure S4, Supporting Information for results from other cell groups). Therefore, with regard to prospective timing, MSNs employed a similar strategy as scalar coding to produce subjective timing.

Building on this hypothesis, we sought to determine if the moving distance of neural trajectories influenced the differentiation between “short” and “long” choices when distinguishing between 900 and 1000 ms in the TBT. We observed that a subset of trials in the 900 ms condition were chosen by the subjects as “long,” while some trials in the 1100 ms condition were chosen as “short.” We hypothesized that longer neural trajectories over a set duration result in a higher speed within the PC space, thus leading to a “long” response (Figure 5E). Consequently, we visualized neural trajectories from both “short” and “long” choice trials in the 900 and 1100 ms conditions (with an example outlined in Figure 5F, where blue signifies short selections and green denotes long). Subsequently, we generated the cumulative moving distance of these trajectories relative to duration and calculated their moving speeds across these conditions. The findings from the MSN group aligned well with our hypothesis. As depicted in Figure 5G,H, the accumulated distances corresponding to “long” choices (green dots) were consistently above those for “short” choices (blue dots) in both the 900 and 1100 ms contexts. Opting for long durations also correlated with faster speeds compared to the short choices in both conditions (insets in Figure 5F,G, *p* < 0.001, paired *t*‐test). Nonetheless, none of the other regions demonstrated this pattern when distinguishing 900 from 1100 ms (Figure S5, Supporting Information), highlighting the critical role of MSNs in identifying short durations within retrospective timing.

### 2.5 D2‐MSNs are the Primary Contributors for Both Retrospective and Prospective Timings

**Figure 6:**
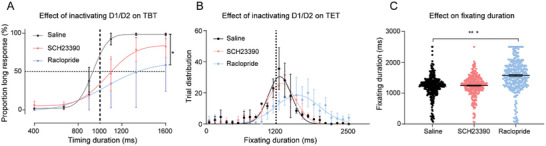
Effects of Inactivating D1/D2‐MSNs on Timing Behavior. A,B) Comparison of behavioral outcomes between control and D1/D2‐MSN inactivation during the TBT (A) and the TET (B). *p < 0.05, two‐way repeated measures ANOVA, followed by Tukey's multiple comparisons test. Error bars indicate SEM. C) Impact of D1/D2‐MSN inactivation on the distribution of fixation durations during the TET. The color coding indicates different treatments: black for saline, red for SCH23390 (D1 receptor antagonist), and blue for Raclopride (D2 receptor antagonist).***p < 0.001, Kruskal‐Wallis test, followed by the Corrected Dunn's Test for Multiple Comparisons.

After emphasizing the crucial function of MSN in timing processes, one question still needed clarification. The presence of two primary cell types—D1‐ and D2‐MSN—in the striatum raised uncertainty regarding which specific type contributed to this particular function. To address this, we conducted experiments to selectively inactivate either D1‐ or D2‐MSN using receptor antagonists, specifically SCH23390 and Raclopride, which have been extensively utilized in previous studies.^[^ ^9^ ^]^ Our findings revealed that inactivating D1‐MSN resulted in only a minor alteration in subjects performance in the TBT and TET, whereas silencing D2‐MSN significantly influenced their subjective timing in both tasks (**Figure** **6**A,B). Notably, the inhibition of D2‐MSN led subjects to make more “short” choices in “long” conditions during the TBT (*p* < 0.05, One‐Way ANOVA) and increased fixation times in the TET. A trial‐by‐trial analysis in the TET indicated that the average fixation duration after administering the D2 antagonist was 1572 ± 25.8 ms, compared to 1219.8 ± 19.5 ms in the saline control group (*p* < 0.001, Kruskal‐Wallis test with Corrected Dunn's Test for Multiple Comparisons, Figure 6C). In contrast, the disruption of D1‐MSN did not produce a similar effect (1246.2 ± 18.6 ms, *p* > 0.05). Consequently, we concluded that D2‐MSN is pivotal in both retrospective and prospective timing functions.

### 2.6 The PFC Coordinates with the Striatum to Ensure Precise Timing

**Figure 7:**
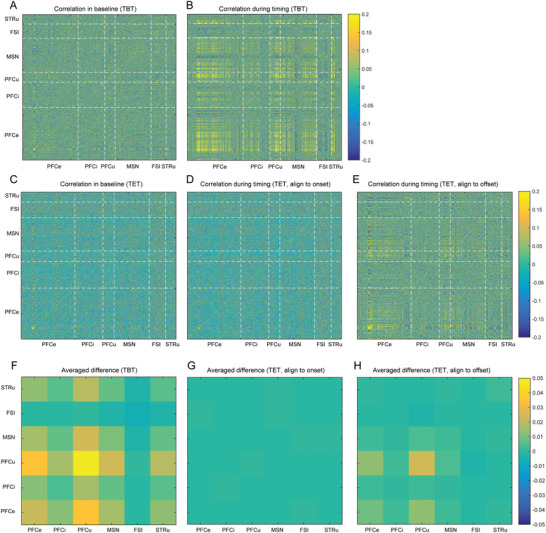
Correlation of Responses Among Different Neuron Groups. A,B) Correlations among individual neurons during the baseline condition (A) and timing in the TBT (B). C‐E) Correlations among individual neurons during the baseline (C) and the timing task in the TET. Panels (D) and (E) present correlations aligned to the onset and offset of the timing intervals, respectively. F) Average correlation between each neuron group during the TBT. G,H) The average correlation between each neuron group during the TET.

Finally, we conducted an in‐depth analysis of regional coordination during timing processing. We calculated the correlation between each neuron's responses prior to and during the timing task, subsequently organizing these correlations by region. By utilizing the correlation coefficient (r) as our metric, we illustrated the variations in the heatmap across regions during timing. Notably, in the TBT, we observed heightened correlations among the majority of neurons, particularly between the PFCe and PFCu, as well as with certain MSN and STRu (**Figure** **7**A,B). By computing the differences between these two critical phases, we produced the differential map shown in Figure 7F, averaging the variations across different cell groups. It was evident that PFCu not only strengthened its correlation with itself but also with nearly all other neuronal populations, with a particularly striking cross‐region correlation between PFCe and MSN. This finding underscores the essential role of the PFC in timing processing and highlights the coordination between striatal MSN and the PFC during retrospective timing.

Similarly, we performed the same type of analysis for the TET, generating a correlation map for all neuronal groups. When assessing correlations based on response profiles aligned with the onset of timing, no significant changes were noted (Figure 7C,D). However, aligning responses to the timing offset revealed enhanced correlations across almost all groups (Figure 7E). The differential maps further indicated that aligning to the onset did not reveal any significant enhancements (Figure 7G), in contrast to aligning to the timing offset, which identified PFCu, PFCe, and MSN with notable correlation increases (Figure 7H). Interestingly, these groups were the same as those observed in the TBT. These results suggest that effective processing—whether retrospective or prospective—necessitates coordination between the PFC and the striatum.

## 3 Discussion

Accurate timing is recognized as a fundamental capability of the brain that underlies various cognitive functions. In conclusion, our research has shown that retrospective and prospective timing utilize different neural mechanisms, thus highlighting the intricate nature of temporal perception in the brain. We emphasized the crucial involvement of MSNs, particularly D2‐MSNs in the striatum, as key facilitators of timing processes. This suggests that focused studies on these neurons could provide valuable insights into the neural foundation of temporal perception and its related behavioral responses. Furthermore, we pointed out the significance of neural coordination and interregional connectivity in timing mechanisms, offering essential revelations about how the brain harmonizes information across diverse cell types and areas to effectively handle the complexities of temporal perception and action.

### 3.1 Retrospective and Prospective Timings are Two Distinct Mechanisms

Our investigation into retrospective and prospective timing has revealed striking differences in the neural dynamics underpinning these two forms of time perception.^[^ ^1^, ^10^ ^]^ The analysis of single‐neuron responses illustrates that the firing characteristics of neurons in both the striatum and prefrontal cortex vary significantly between tasks requiring retrospective versus prospective timing. Specifically, our data indicate that neurons exhibit distinct temporal firing patterns that align with the cognitive demands of each timing task.

In prospective timing, where participants anticipate the duration of an event, we observed increased neuronal firing rates leading up to the expected interval for a substantial of neurons, which is in line with previous studies.^[^ ^2^, ^3^, ^4^ ^]^ This anticipatory activation may reflect the neural encoding of the predicted duration and the preparatory processes required to execute a forthcoming response. For example, in the prefrontal cortex, a higher portion of PFCe and PFCu neurons showed peak responses during the countdown phase in the TET than in the TBT (Figure 2D–F and Table 1), highlighting the role of attentional mechanisms and executive control.^[^ ^11^ ^]^ Conversely, during retrospective timing tasks, where participants assess the duration of a previously experienced event, neuronal firing patterns were notably delayed and tended to peak after the completion of the interval (Figure 2D–F). This suggests that the neural representation of temporal information is not only influenced by the encoding of time but also by the retrieval processes involved in memory assessment.^[^ ^12^ ^]^

Furthermore, the cross‐region correlation provided compelling evidence of different neural ensembles being recruited for each timing task. In retrospective timing, we observed broad synchronous firing between the PFC and the striatum. In contrast, the cross‐region correlations during prospective timing were more limited to the PFC (Figure 7; Figure S6, Supporting Information). This finding underscores the retrospective reevaluation of duration as a more complex process, potentially involving the reconsolidation of memories and the broader contextual processing of past experiences.^[^ ^12^ ^]^

Collectively, our findings suggest that retrospective and prospective timing engage distinct neural mechanisms, spotlighting the complexity of temporal perception within the brain. These differences reflect not only the separate cognitive and neural demands of each type of timing task, but also hint at broader implications for how the brain constructs and utilizes temporal information in decision‐making and memory formation.^[^ ^13^ ^]^ Future investigations should delve deeper into these neural dynamics, exploring how they may inform our understanding of time perception and its potential disruptions in various cognitive disorders.

### 3.2 The Importance of MSNs in Temporal Processing

Our findings reveal a significant distinction in the roles of different neuronal populations in the striatum concerning temporal processing, highlighting the pivotal contribution of MSNs over FSIs and prefrontal neurons.^[^ ^2^, ^3^, ^14^ ^]^ Notably, D2‐MSNs emerge as the primary contributors to both retrospective and prospective timing (Figure 6), marking them as critical players in the encoding and retrieval of temporal information.^[^ ^14^, ^15^ ^]^

The prominence of D2‐MSNs in temporal processing can be linked to their unique neurochemical properties and synaptic connectivity. These neurons are known for their role in modulating reward‐based behaviors and regulating the overall output of the basal ganglia circuitry. Our data suggest that D2‐MSNs may enhance the precision of temporal estimates by integrating incoming signals about duration and facilitating the weighting of predictive cues. This ability is particularly pronounced in retrospective timing, where the evaluation of past intervals may require a nuanced interpretation of temporal memory.^[^ ^12^ ^]^

In contrast, the lesser role of D1‐MSNs in this context is intriguing. While it is well‐established that D1‐MSNs are integral to movement initiation and reinforcement learning,^[^ ^16^ ^]^ their contribution to timing tasks appears more secondary. This finding suggests that distinct neural circuits within the striatum play specialized roles depending on the timing context, with D2‐MSNs potentially serving as a dedicated mechanism for temporal processing.

To further clarify the functional difference between D1‐ and D2‐MSNs, we envision several future experiments utilizing the pharmacological approach: 1) local administration of antagonists directly to the striatum; 2) additional behavioral assessments to evaluate the influence of D1/D2‐MSN modulation on anxiety, motivation, and other timing‐related activities; and 3) simultaneous recording of neuronal activity alongside behavioral observations. These proposed experiments should significantly enhance the understanding of the functional dynamics of D1‐ and D2‐MSNs in the context of timing processes.

Furthermore, the reduced involvement of FSIs highlights the importance of the principal output neurons over local inhibitory circuits in this specific cognitive function. While FSIs undoubtedly play a role in modulating broader neural activity and enhancing signal‐to‐noise ratios, their contribution to the intricate computations needed for accurate timing appears limited when compared to the dedicated functions of MSNs.

### 3.3 The Importance of the Coordination Within PFC and Between PFC and Striatum

Our findings indicate that the timing process is characterized by enhanced coordination among different cell types within the prefrontal cortex, as well as a strengthened correlation between the prefrontal cortex and the striatum (Figure 7). This enhanced synchrony suggests a refined neural network configuration that optimally supports temporal processing. The prefrontal cortex, well‐known for its role in executive functions and decision‐making, appears to orchestrate the activity of varied neuronal populations during timing tasks. This coordinated firing may facilitate a more integrated representation of temporal information, enabling the accurate prediction and assessment of intervals.

The observed enhancement in firing synchrony across diverse cell types within the prefrontal cortex indicates a potential mechanism for the increased robustness of temporal coding. Such synchrony could enhance the reliability of neuronal signals by amplifying specific temporal cues while suppressing noise. This precision in timing may be crucial for distinguishing between intervals, particularly in complex tasks where multiple timing cues are presented concurrently. Enhanced synchrony may also support the modulation of attention,^[^ ^17^ ^]^ allowing for a more effective allocation of cognitive resources to relevant temporal information.

Additionally, our data highlight a strengthened correlation between the prefrontal cortex and the striatum during the timing process. The striatum, involved in reward processing and motor planning,^[^ ^16^, ^18^ ^]^ plays a fundamental role in translating temporal estimates into action. The observed increase in connectivity during timing tasks suggests that temporal information is not only encoded in isolation within the prefrontal cortex but is also shared with the striatum for downstream impact on behavior.^[^ ^2c,d,i^ ^]^ This collaborative network likely supports the integration of temporal assessment with decision outcomes, enhancing both timing precision and action execution.

## 4 Experimental Section

### Animals

Two Rhesus macaques (monkey 1, weighing 8.3 kg and aged 6.8 years; monkey 2, weighing 9.2 kg and aged 9 years) were trained to perform two interval timing tasks. The monkeys were housed individually in a facility accredited by AAALAC, which maintained controlled temperature and humidity levels (humidity: 40–70%; temperature: 20–26 °C).^[^ ^19^ ^]^ Each colony room operated on a 12:12‐h light‐dark cycle. To facilitate head fixation, all monkeys were surgically implanted with a titanium pillar and were trained to remain seated calmly in a chair while performing behavioral tasks. Prior to the formal collection of experimental data, all monkeys underwent a minimum of four months of training, allowing them to become accustomed to the three facility staff members involved in the study. All procedures adhered strictly to the National Institutes of Health Guide for the Care and Use of Laboratory Animals and received approval from the Institutional Animal Care and Use Committee (IACUC) at the Shenzhen Institute of Advanced Technology, Chinese Academy of Sciences (approved ID: SIAT‐IACUC‐201123‐NS‐DJ‐A1483).

### Behavioral Task—Temporal Bisection Task (TBT)

The TBT was adapted from earlier research conducted on rodents.^[^ ^2^, ^3^, ^20^ ^]^ Each trial commenced with the presentation of an indicative stimulus, a yellow dot, positioned at the center of the screen (Figure 1A; Video S1, Supporting Information). When the monkey maintained fixation on this yellow dot for a variable duration ranging from 0.5 to 1 s, the dot transformed into a white square, signaling the start of the timing phase. The white square was displayed for varying lengths of time (0.4, 0.67, 0.9, 1.1, 1.33, and 1.6 s), during which the monkeys were required to keep their fixation on it until it disappeared. Following the disappearance of the white square, a green disc and a red disc appeared simultaneously in the upper left and lower right corners of the monitor, respectively. The monkeys then had to determine whether the duration of the presentation was longer or shorter than 1 s by making a saccade to one of the two discs, which were located 7.3 visual degrees from the center and distinguished by their colors (green and red, as illustrated in Figure 1A). A correct response would earn the monkeys a juice reward. If the subject broke fixation (including blinks), looked outside the designated fixation area, or failed to make a choice within 3 s, the trial would be terminated immediately and classified as incomplete. Each TBT session consisted of 600 trials, with each duration being presented 100 times.

### Behavioral Task—Temporal Estimation Task (TET)

The TET was adapted from earlier research conducted on animals.^[^ ^2^, ^3^ ^]^ Each trial commenced with a fixation point displayed at the center of the screen, where subjects were required to maintain their gaze for 0.5 s (Figure 1D; Video S2, Supporting Information). Following this, an indicative stimulus—a yellow square—appeared in one of four positions on the screen (top left, bottom left, top right, or bottom right). The monkey was then required to fixate on the yellow square for a variable duration ranging from 0.5 to 1 s, after which the square would change to blue, signaling the start of the timing phase. During this timing phase, the monkey needed to keep its fixation on the blue square and break the fixation between 1.2 and 2.4 s. The monkey would receive a drop of juice as a reward after making a correct response. A trial would automatically terminate if the monkey failed to maintain fixation on the initial point, the indicative stimulus, or broke fixation earlier than 1.2 s or later than 2.4 s. Each TET session comprised 300 trials.

### Experimental Procedure

During the experiments, the monkeys were securely seated in a chair, their heads fixed in a forward position toward an LED monitor (AUSU, 24 inches, 1920 × 1080 resolution, 144 Hz refresh rate) positioned 57 cm away. A flexible tube was attached to the monkeys mouths to administer juice as a reward. Their gaze positions and pupil size were continuously tracked and recorded using an eye‐tracking system (Eyelink 1000 Plus, with a sampling rate of 1000 Hz). A nine‐point calibration was conducted to ensure accurate measurement of the subjects' eye positions. All experiments took place in a controlled environment that was both dark and quiet. A Matlab‐based toolbox, MonkeyLogic (NIMH version), was used for experimental control and coordinating behavioral and electrophysiological signals. To mitigate any potential bias in the animals’ behavioral performance, the experiments were consistently initiated ≈10:00 am, continuing for 3 to 4 h each day.

### Electrophysiological Recording

A semi‐chronic micro‐drive with 128 electrodes (128FL‐42 mm, Gray Matter Research) was installed on each monkey to enable in‐vivo electrophysiological recording from the prefrontal cortex and striatum. Such a micro‐drive allows flexible adjustment of the recording depth for each electrode.^[^ ^21^ ^]^ Typically, an electrode was advanced slowly by tuning the driving screw in a step of 1/4 to 1/2 turn (8 turns/mm). During the recording, signals were collected using a 128‐channel recording system (alpha‐RS, Alpha Omega) at a sample rate of 30K Hz and then amplified and bandpass filtered (250–8000 Hz). Spikes were identified in real‐time by manually setting thresholds. After the recording, spiking signals collected online were further processed by the widely used sorting software, Offline Sorter (Plexon Inc, Dallas). In brief, after removing noises and abnormal waveforms, spikes were sorted automatically using the K‐means clustering method. The sorted spikes were then used for further analysis using custom MatLab codes.

### Classification of Neuron Groups in the Striatum and Cortex

It successfully identified a total of 3511 neurons in this study. The corresponding waveforms and their respective recording locations are illustrated in Figure 1I. Among these neurons, 1333 were identified in the striatum, while 1944 were in the prefrontal cortex. Following the methodology established in previous research,^[^ ^8^ ^]^ it applied a nonlinear dimensionality reduction technique known as WaveMAP, followed by Louvain clustering, to the spiking waveforms of these neurons. This approach allowed us to condense the waveform data into a two‐dimensional format, which was then categorized using the Louvain clustering method. This analysis was conducted separately for neurons in the striatum and cortex.

The striatal neurons were classified into three distinct clusters. For each cluster, it assessed three key features of the neurons' waveforms: action potential (AP) width, peak ratio, and trough‐to‐peak duration.^[^ ^8^, ^18^, ^22^ ^]^ Additionally, it considered the firing rate for each cluster. Notably, one cluster exhibited a significantly shorter trough‐to‐peak duration, narrower AP width, higher peak ratio, and the highest firing rate, leading us to hypothesize that these neurons were putative fast‐spiking interneurons (FSI). In contrast, another cluster displayed the lowest peak ratio and the longest AP width duration, suggesting that these neurons may correspond to medium spiny neurons (MSN).^[^ ^22^, ^23^ ^]^ The rest cluster was classified as unidentified (STRu).

Prefrontal neurons were also categorized into three clusters. The first cluster displayed the shortest trough‐to‐peak durations, the highest peak ratio, and the lowest AP width, leading us to speculate that these neurons were putative inhibitory neurons (PFCi). The second cluster exhibited longer trough‐to‐peak durations, lower firing rates, and extended AP width durations, suggesting they may represent cortical excitatory neurons (PFCe). The last cluster did not demonstrate significant differences in waveform characteristics, and thus, was labeled as unidentified PFC (PFCu).

### Verification of Electrode Localization

The locations of the microdrive electrodes were verified through a combination of preoperative anatomical MRI scans (obtained prior to the microdrive implantation), postoperative MRI scans, computed tomography (CT) scans, and detailed tracking notes documenting the depths of the electrodes.

Initially, the tip of each electrode was positioned above the dura pointing to the frontal lobe or the striatum. As the microdrive advanced, electrodes slowly passed from the superficial layer of the cortex to deeper sites. To reconstruct the coordinates of each neuron's recording site, three specific coordinates were manually identified: x‐ and y‐ positions to define the location on the horizontal plane, which were confirmed by post‐implantation MRI scans, and the depth (z‐) calculated from the tracking notes for each recording session. A visual inspection of the pre‐operative MRI images further confirmed the location of the recording sites for each monkey. At the end of the experiment, CT scans were conducted on the monkeys to verify the electrode trajectories by overlaying them on the pre‐operative MRI images. For visualization purposes, the waveform of each neuron was illustrated in a 3‐D space (Figure 1), based on the respective coordinates relative to the electrodes.

### Behavioral Data Analysis

Curve fitting and parameter estimation were conducted utilizing Prism software (Version 5; GraphPad Software Inc., San Diego). A Gaussian cumulative function (Equation 1) was employed to model the present duration and *P_L_* obtained from the TBT.

(1) $$F\;\left(t\right)=a+\frac{b}{\sigma \sqrt{2\pi }}\int_{-\infty }^{t}\left[exp-\left(\frac{{\left(t-\mu \right)}^{2}}{2\sigma^{2}}\right)\right]dt$$

In this function, *F*(t) denotes the *P_L_* corresponding to a specific duration *t*, *µ* signifies the mean, and *σ* indicates the standard deviation, which reflects the slope of the function. The parameter *a* represents the lower asymptote, while *b* indicates the range of the function. The mean *µ* was also identified as the Point of Subjective Equality (PSE), defined as the point at which there was a 50% probability that the subject will respond with a “long” duration (*P_L_* = 50%). This metric captures the subjectively perceived duration of time, with variations interpreted as underestimations or overestimations of the actual duration.^[^ ^24^ ^]^ The goodness of fit for this function was evaluated using R^2^.

Another Gaussian function (Equation 2) was applied to fit the anticipated trial number and timing duration in TET.

(2) $$F\left(t\right)=a+\frac{b}{\sqrt{2\pi \sigma }}\left[exp-\left(\frac{{\left(t-\mu \right)}^{2}}{2\sigma^{2}}\right)\right]$$

In this function, *F(t)* denotes the anticipated number of trials within a bin corresponding to a specific sample *t*, where *t* signifies the timing duration. The parameter *a* indicates the lower asymptote, which was set at 0, while *b* represents the maximum expected number of trials for this distribution. Additionally, *σ* and *µ* were used to denote the variance and mean of the distribution, respectively.

### Classification of Neurons Peaking at Different Phases

The objective of this analysis was to determine the number of neurons that exhibit peak activity during the timing process. In the TBT experiment, it collected Peri‐Stimulus Time Histograms (PSTH) from 1000 ms prior to the onset of timing until 2600 ms after it, using 1600 ms trials with a 20‐ms bin size, smoothed by sliding windows. The PSTH was normalized based on the values from 1000 to 500 ms before the timing began. A neuron was classified as peaking during the timing process if it met two criteria: 1) the maximum value of the normalized PSTH exceeded 0.6 during the timing phase,^[^ ^2a^ ^]^ and 2) the peak position fell within the timing interval.

In the TET experiment, it included trials with a timing duration ranging from 1000 to 2400 ms in our analysis. PSTH data were gathered from 1000 ms before the timing onset to 2900 ms post onset, and normalized using the baseline 1000 to 500 ms prior to the timing onset. The timing range was defined from the onset of timing to the mean timing duration across all trials. The criteria for determining whether a neuron peaks during the timing process remained consistent with those used in the TBT analysis.

### Classification of “Scalar” and “Absolute” Coding Neuron

The criteria for classifying a neuron as a “Scalar” or “Absolute” coding neuron have been adapted from earlier research.^[^ ^2^, ^4^ ^]^ This analysis aims to assess the degree of similarity in a neuron's response profiles across varying timing durations. Specifically, it seeks to determine whether the response profile for a short timing duration was a subset of or a compressed version of the profile for a longer timing duration.

To achieve this, it first calculated the PSTHs for all timing durations. It designated the longest PSTH (for example, the one derived from 1600 trials in the TBT) as the LONG, while the others were categorized as SHORTs. Subsequently, it either compressed or truncated the LONG PSTH to match the length of each SHORT and conducted pairwise comparisons between the modified LONG and each SHORT. The similarity between these comparisons was quantified using Pearson correlation coefficients and Euclidean Distance (as outlined in Equation 3).

(3) $$Dist=\sum_{t=1}^{n}\begin{array}{c} \;\\ \sqrt{{\left(\mathrm{PSTH}1\left(t\right)-\mathrm{PSTH}2\left(t\right)\right)}^{2}} \end{array}\;$$

In this context, “n” denotes the length of a specific PSTH, PSTH1 refers to one of the SHORTs being compared, and PSTH2 indicates the truncated or compressed LONG. A neuron was classified as a “Scalar neuron” if: 1) the Pearson correlation coefficients between the compressed LONGs and SHORTs exceeded 0.3, and 2) the average Euclidean Distance between the compressed LONGs and SHORTs was less than that between the truncated LONGs and SHORTs. Conversely, a neuron was identified as an “Absolute neuron” if: 1) the Pearson correlation coefficients between the truncated LONGs and SHORTs were all greater than 0.3, and 2) the average Euclidean Distance between the truncated LONGs and SHORTs was shorter than that between the compressed LONGs and SHORTs.

### PCA and neural trajectory construction in the TBT and TET

The methodology of Principal Component Analysis (PCA) applied to the timing process was based on prior research.^[^ ^6^ ^]^ In the TBT, it aimed to uncover the dynamic latent structures present in the timing processes across six different durations, ranging from 400 to 1600 ms. To achieve this, it first constructed a timing matrix with dimensions [neuron × time] for each of the six durations. Subsequently, it concatenate these six timing matrices along the temporal dimension, resulting in a comprehensive concatenated timing matrix with dimensions [neuron × (400 + 670 + 900 + 1100 + 1330 + 1600)]. It then applied PCA to this matrix to extract the principal components (PCs) corresponding to all timing durations, projecting the top three PCs into a PC space to construct the neural trajectories.

To further explore the dynamic latent structures during the timing process of the TET, it focused on trials with timing durations ranging from 1000 to 2400 ms. It created a timing matrix that represented the average PSTH for each neuron from the onset of timing up to 1000 ms (align to timing onset) and from 1000 ms before the timing offset to the offset of timing (align to timing offset). These two matrices were then concatenated along the temporal dimension, followed by PCA to derive the PCs that represented a low‐dimensional view of the timing process.

To elucidate the neural trajectories during the TET timing process across varying lengths, it categorized all trials into four distinct ranges based on the timing duration for each neuron (Range 1–4). The timing durations for Range 1–4 were 1000–1558, 1108–1773, 1226–1986, and 1348–2378 ms, respectively. It then computed the average PSTH from the timing onset to the shortest timing duration post‐onset for each range. For instance, for neurons in Range 2, it constructed a timing matrix using the PSTH from the timing onset to 1108 ms (20 ms bin width). This resulted in four separate timing matrices. Finally, it concatenated these matrices along the temporal dimension and performed PCA on the combined data (aligned to timing onset). However, it was important to note that this approach may lead to a loss of information regarding neural activity close to the timing end. To address this, it employ a similar method to create four neural trajectories aligned to the timing offset, capturing data from the shortest timing duration before the timing offset to the offset (aligned to timing offset).

### Comparing the Speed of Neural Trajectories in the TET

To uncover the relation between neural trajectories and the subjective timing estimation in the TET, it analyzed the speed of neural trajectories and compared with subjects timing duration. For this purpose, it separated trials into five ranges (see previous section) and created neural trajectories for each of them using primary PCs that accounted for at least a 70% contributing rate (varied from 6 to 19 PCs for different neuron groups). The cumulative Euclidean distance was plotted in a 20‐ms resolution against time for each range. Next, it calculated the average speed of each trajectory, and then it calculated the Pearson correlation coefficient between the speed and mean fixate duration of each range. This analysis was applied to all neuron groups and particularly for scalar coding neurons.

### Comparing the Speed of Neural Trajectory in the TBT

To analyze the speed of neural trajectories in the TBT, it aimed to uncover the underlying variables influencing short and long choices within the 900 and 1100 ms conditions. Considering that monkeys tended to favor short choices in the 900 ms condition and fewer short choices in the 1100 ms condition, it employed a Bootstrapped approach to balance the trial numbers for both choice types. Our analysis was limited to neurons that recorded a minimum of 10 long choices in the 900 ms condition or 10 short choices in the 1100 ms condition.

For each neuron, it selected 10 trials each for short and long choices to create a PSTH, thereby constructing a timing matrix for both choice types (PSTH × neuron). Subsequently, it conducted PCA on these two timing matrices concurrently to identify the principal components (example neural trajectories can be seen in Figure 5F). The speeds of the neural trajectories were calculated using the primary PCs that accounted for at least a 70% contributing rate (5 – 6 PCs). This process was repeated 2000 times, resulting in 2000 paired speeds for both short and long choices. To determine statistical significance, it employed a paired *t*‐test.

### Calculating the Interneuronal Correlations Among Neuron Groups

In order to assess the interneuronal correlations for different neuron groups, it computed the Pearson correlation coefficient between the PSTH of each neuron during the baseline period or when timing occurred. For the TBT, trials from the 900 ms condition were incorporated into the analysis. The response that occurred 900 ms prior to the onset of timing was designated as the baseline.

In the TET, trials with a timing duration ranging from 1000 to 2400 ms were included in the analysis. PSTHs from 1000 ms after the onset of timing (aligned to the timing onset) or 1000 ms before the offset of timing (aligned to the timing offset) were employed to evaluate the interneuronal correlations during the timing period. The PSTH from 1000 ms before the onset of timing was used to calculate the interneuronal correlations during the baseline.

### Pharmacological Manipulation

Raclopride, a non‐selective antagonist of D2‐like receptors, and SCH23390, a non‐selective antagonist of D1‐like receptors, were employed as pharmacological tools to disrupt the neural activity associated with these receptors, primarily found in MSNs. The dosage of 0.08 mg kg^−1^ and the injection volume of 0.1 ml kg^−1^ were based on prior pharmacological research,^[^ ^9^ ^]^ which demonstrated their efficacy in influencing cognitive performance in rhesus macaques.

All drugs were freshly prepared on the day of experimentation and administered via intramuscular injection in the hind limb. Injections were conducted 15 min prior to the formal testing, during which the monkeys completed a TBT and a TET. Previous studies have indicated that the effects of intramuscular SCH23390 and Raclopride on macaque behavior persist for ≈80 min.^[^ ^9^ ^]^ To ensure optimal drug efficacy, if a monkey was unable to complete both tasks within 80 min post‐injection, that session would be terminated, and a subsequent injection session would be scheduled. A washout period of at least five days was maintained between each injection.

## Conflict of Interest

The authors declare no conflict of interest.

## Author Contributions

X.L., Z.Z., and J.D. performed conceptualization and methodology. X.L., Z.Z., L.G., P.Y., and J.D. performed investigation. X.L. performed formal analysis and visualization. X.L. wrote the original draft. J.D. wrote, review and edited the final manuscript. X.L. and J.D. performed funding acquisition.

## Supporting information



Supporting Information

Supplemental Video 1

Supplemental Video 2

## Data Availability

The data that support the findings of this study are available on request from the corresponding author. The data are not publicly available due to privacy or ethical restrictions.
