# Supplementary material for: Medium Spiny Neurons Mediate Timing Perception in Coordination with Prefrontal Neurons in Primates
Source: Adv Sci (Weinh). 2025 Feb 11;12(16):2412963. doi: 10.1002/advs.202412963 (PMC12021029; doi:10.1002/advs.202412963)
Supplement: Supplementary file 1 — Supporting Information [file ADVS-12-2412963-s003.docx]

**SUPPLEMENTARY INFORMATION**

**Supplementary Videos**

**Supplementary Video 1. Video illustration of the Temporal bisection task (TBT).**

**Supplementary Video 2. Video illustration of the Temporal estimation task (TET).**

**Supplementary Figures**

**
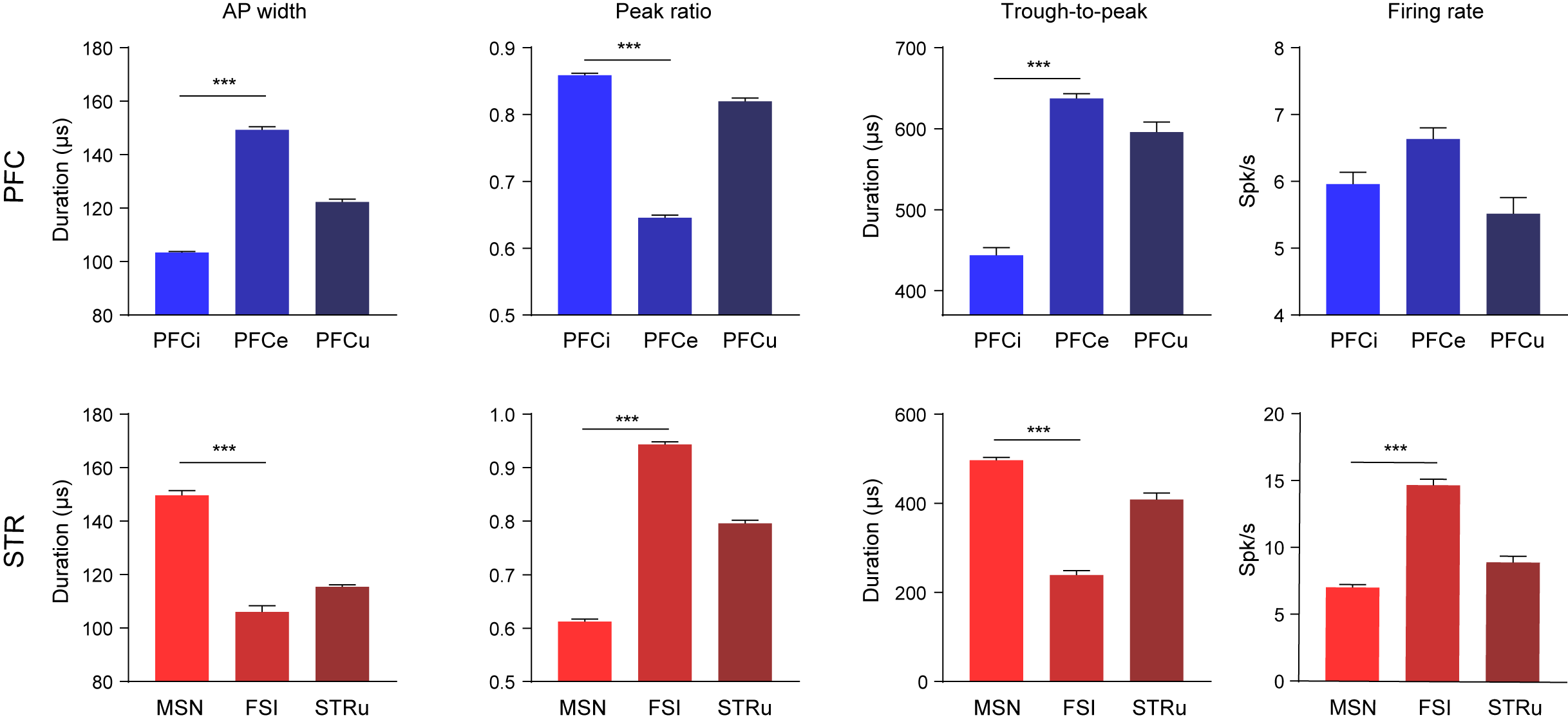
**

**Supplementary Figure S1. Spatial distribution of absolute coding neurons in the TBT and scalar coding neurons in the TET.**

**
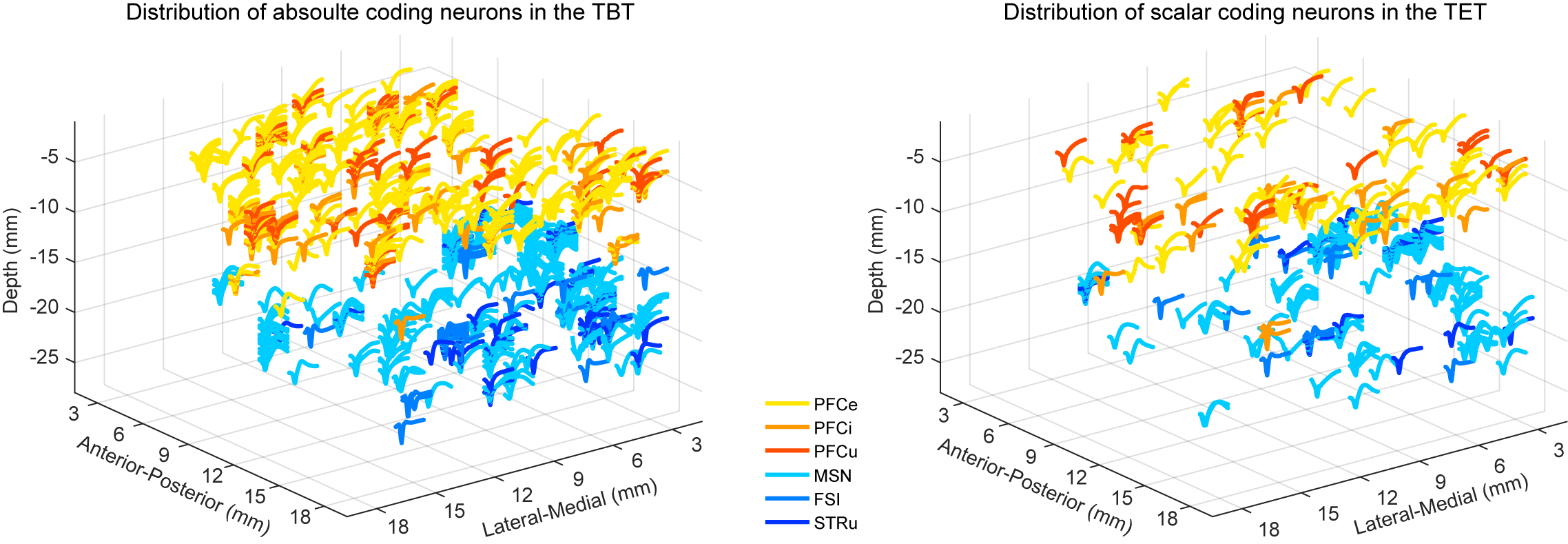
**

**Supplementary Figure S2. Spatial distribution of absolute coding neurons in the TBT and scalar coding neurons in the TET.**

**
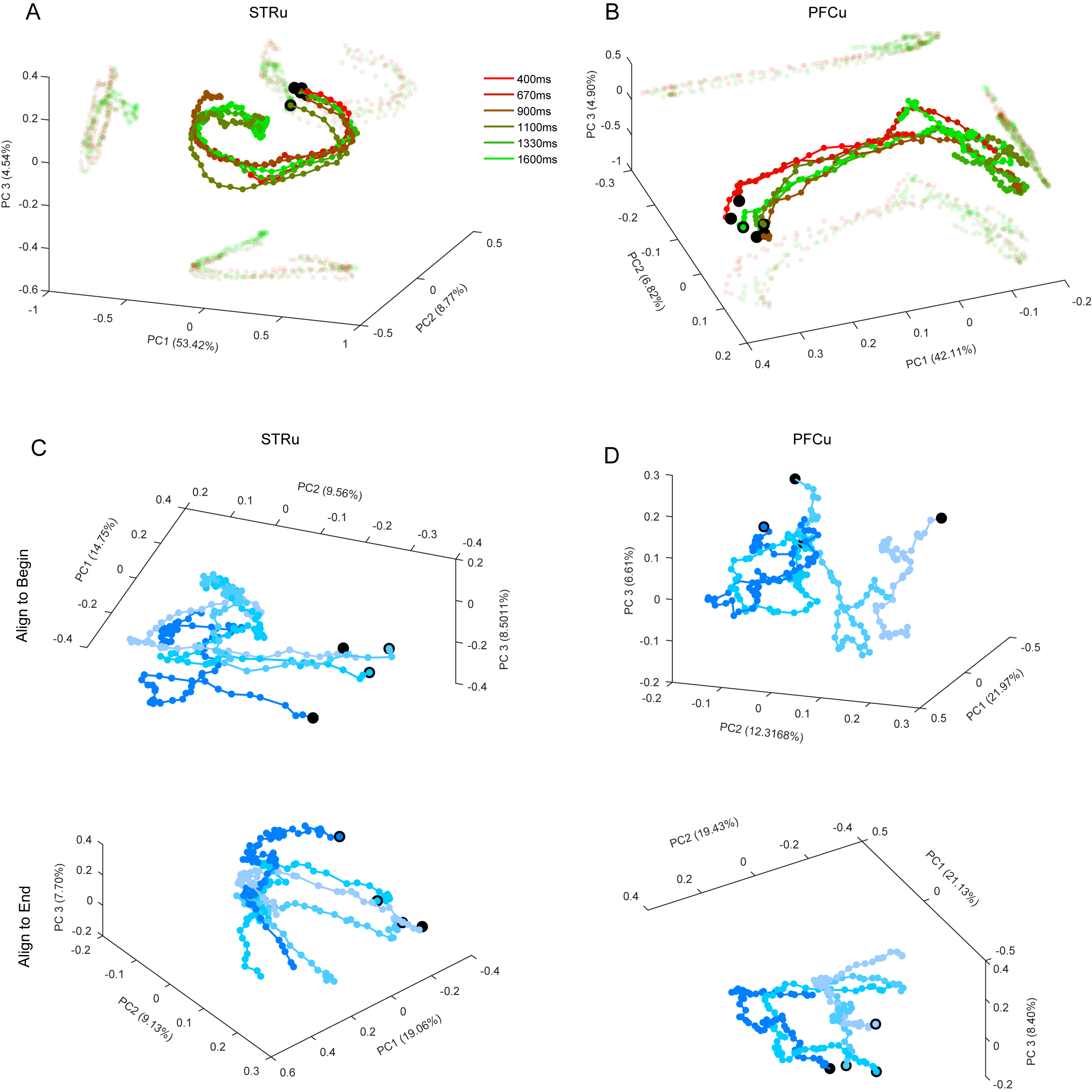
**

**Supplementary Figure S3. Neural trajectories of unidentified neurons in the striatum and PFC.** (A-B) Neural trajectories of STRu and PFCu for six timing durations in the TBT. (C-D) Neural trajectories of STRu and PFCu for six timing durations in the TET.

**
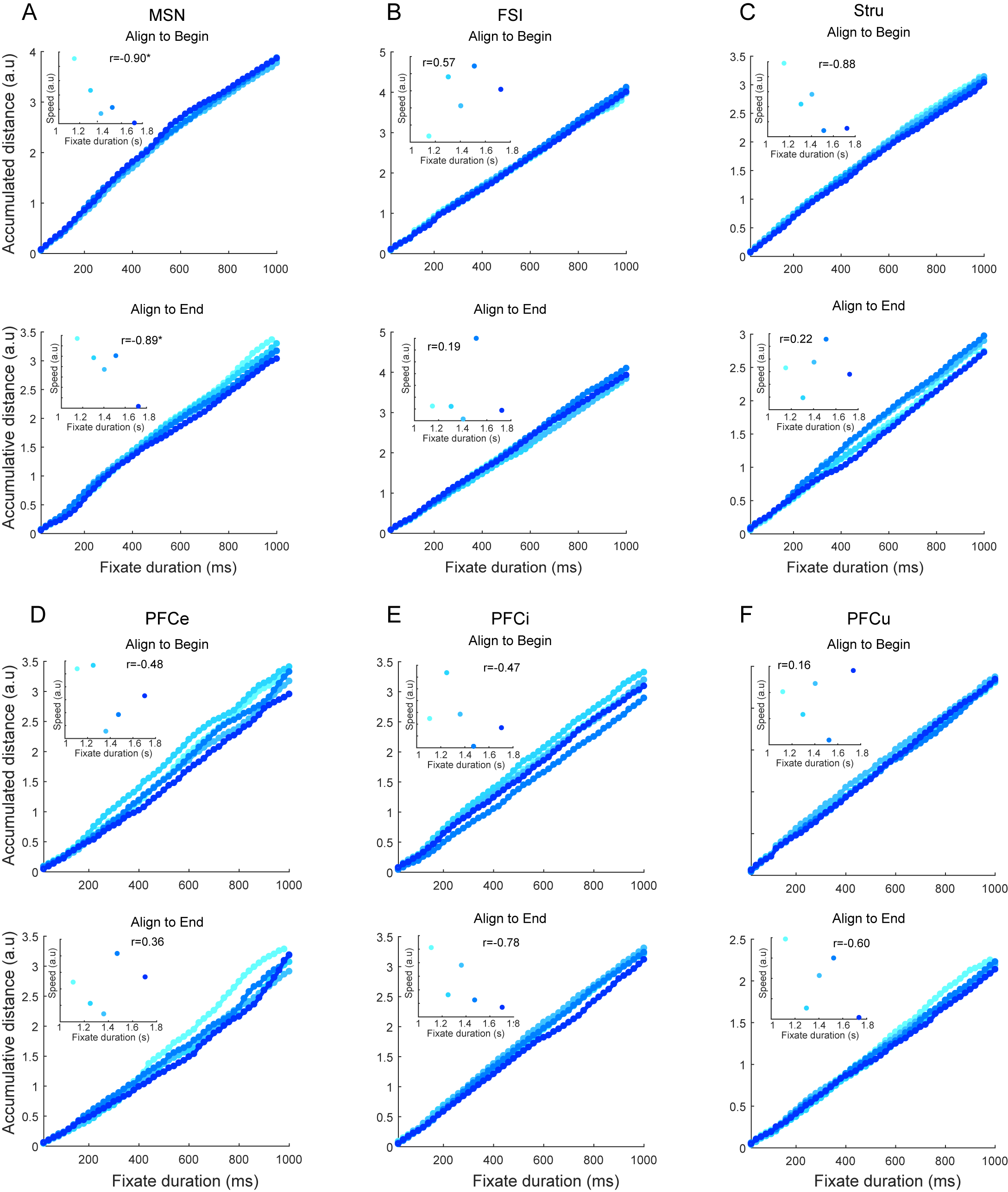
**

**Supplementary Figure S4. The accumulative moving distance of neural trajectories for different neuron groups in the TET.** The insets show the correlation between subjective timing and moving speed. Colors indicate the range of subjective timing.

**
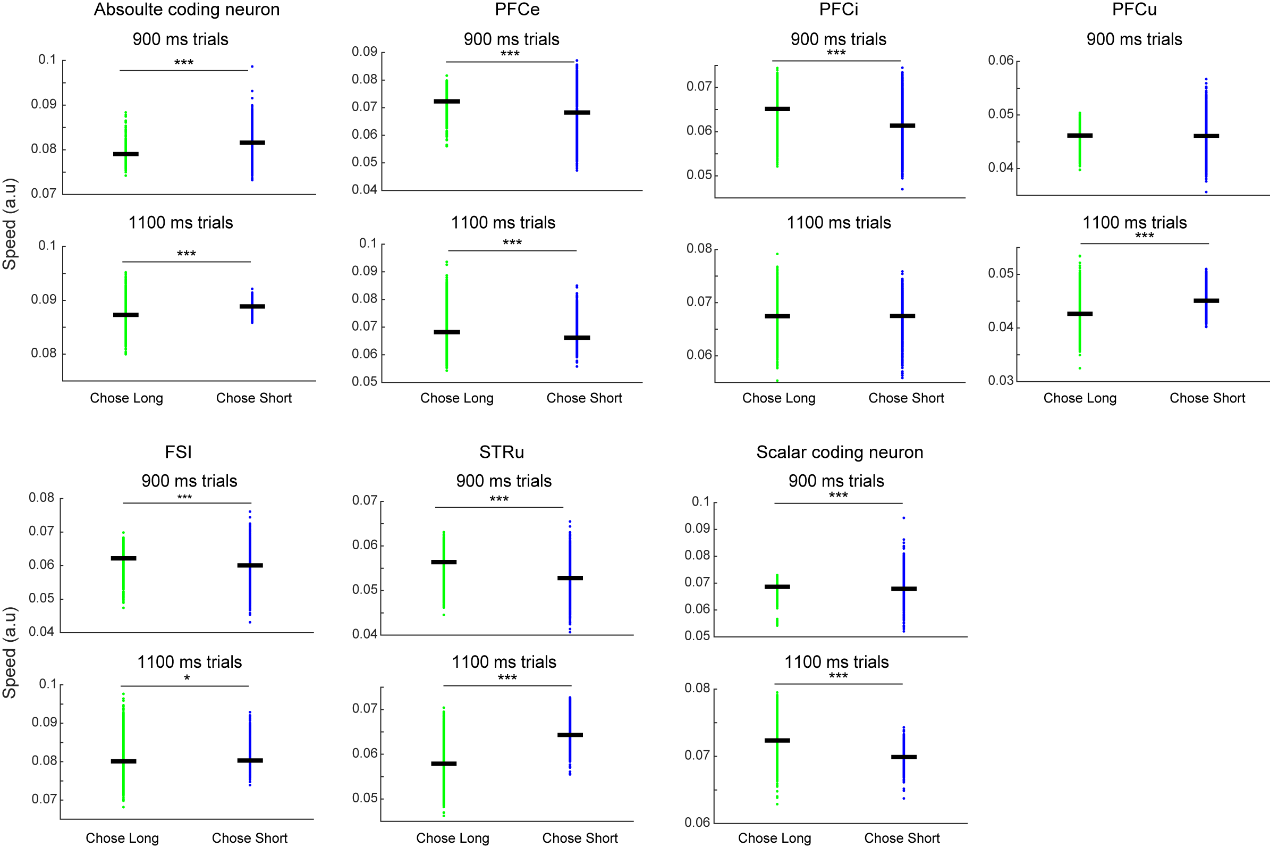
**

**Supplementary Figure S5. The moving speed of neural trajectories in the long and short choice trials for different neuron groups.** Top row: 900 ms condition; bottom row: 1100 ms condition. Green: chose long; blue: chose short. ****p*<0.001.


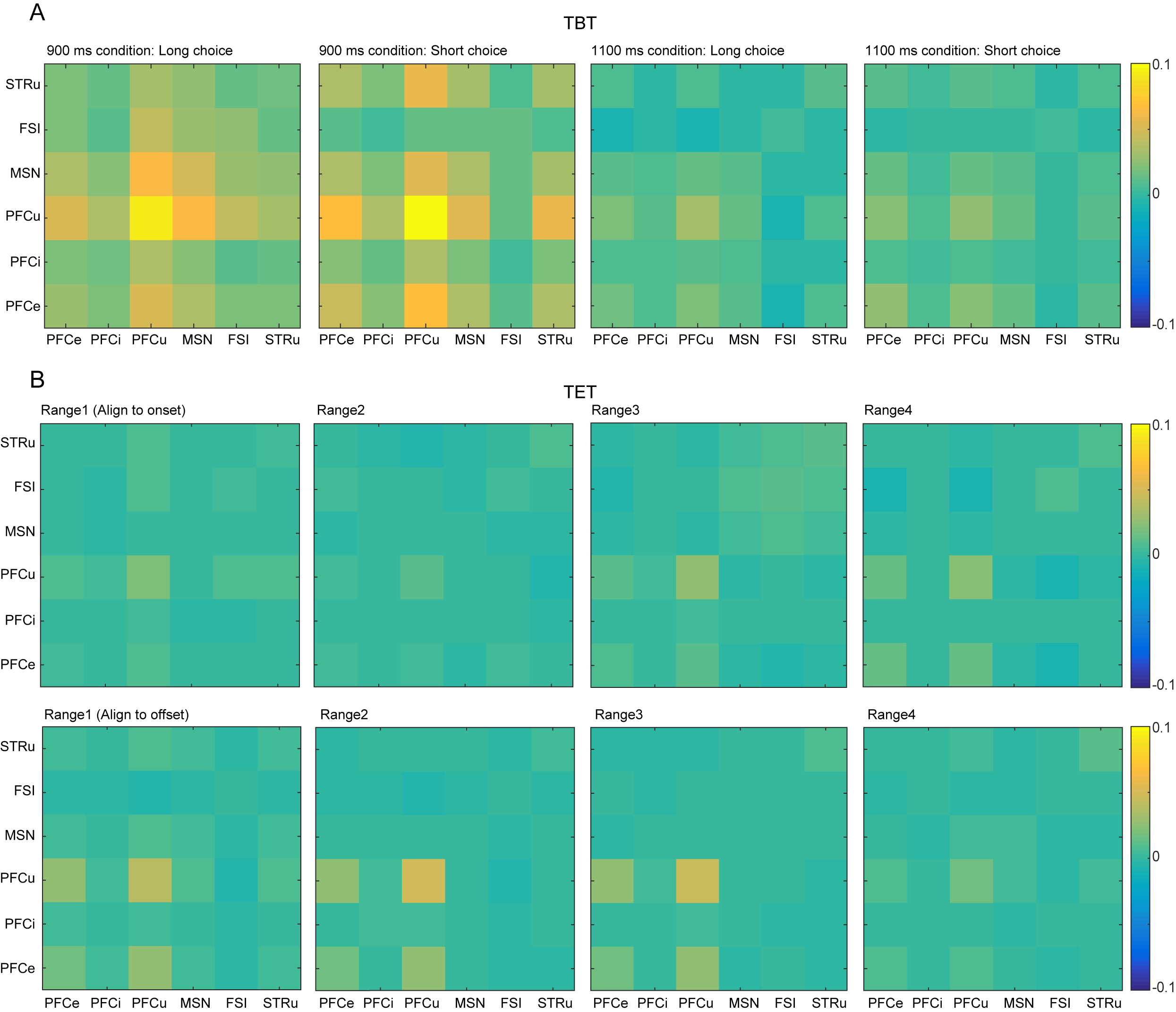


**Supplementary Figure S6. Correlation among different neuron groups in different subjective timings.** (A) The correlation among different groups for the long and short choices, respectively, in the 900 and 1100 ms conditions of the TBT. (B) The correlation among different groups for different timing ranges in the TET. The top and bottom rows were generated by aligning to the onset and offset of timing.
